# Verses

**Published:** 1907-02

**Authors:** 


### VERSES

### THE ODDEST THING.

"Like a man without a wife,
Like a ship without a sail,
But the oddest thing in life,
Is a short without a—proper length."—*Review.*

### THE MORNING AFTER.

Those dry Martinis were too much for me,
Last night I really felt immense,
Today I feel like thirty cents;
It is no time for mirth and laughter
In the cold gray dawn of the morning after.

—*Exchange.*

### TO ALL WOMANKIND.

A bumper to womankind, clumsy or thin,
Young or ancient—it weighs not a feather;
So fill a pint bumper—nay, fill to the brim,
And let's toast 'em, e'en all together.

### WHY HE DIDN'T LIKE THE DOCTOR.

I do not like you, Doctor Fell.
The reason why I can not tell,
But I don't like you, Doctor Fell.

You know the reason very well.
You owed a bill; he made you shell.
That's why you don't like Dr. Fell.

—*Clinical Medicine.*

### OLD JACK FROST.

Ole Jack Frost am now at han'
  Een him cote ob w'ite,
Spedin' heself ober de lan'
  An' chillin' t'ings een site.

He's friz de ponds all ober
  An' edged de streams wid ice.
Hit's cole, sho, but moreober
  Hit looks purty an' nice.

But hit gits yer een de toes,
  An' cools yer down de back,
An' nips yer yeres an' nose
  An' whar youse weak an' slack.

So I wants nun er hit een mine;
  Gimme de summer glow,
Wid blazin' hot sunshine
  Instid de cole an' snow.—B. H. Teague.

